# Distinct disease features of acute and persistent genotype 3 hepatitis E virus infection in immunocompetent and immunosuppressed Mongolian gerbils

**DOI:** 10.1371/journal.ppat.1011664

**Published:** 2023-09-13

**Authors:** Sakthivel Subramaniam, Rafaelle Fares-Gusmao, Shinya Sato, John M. Cullen, Kazuyo Takeda, Patrizia Farci, David R. McGivern

**Affiliations:** 1 Laboratory of Molecular Virology, Division of Emerging and Transfusion Transmitted Diseases, Office of Blood Research and Review, Center for Biologics Evaluation and Research, U.S. Food and Drug Administration, Silver Spring, Maryland, United States of America; 2 Hepatic Pathogenesis Section, Laboratory of Infectious Diseases, National Institute of Allergy and Infectious Diseases, National Institutes of Health, Bethesda, Maryland, United States of America; 3 Department of Population Health and Pathobiology, College of Veterinary Medicine, North Carolina State University, Raleigh, North Carolina, United States of America; 4 Microscopy and Imaging Core Facility, Center for Biologics Evaluation and Research, U.S. Food and Drug Administration, Silver Spring, Maryland, United States of America; The Research Institute at Nationwide Children’s Hospital, UNITED STATES

## Abstract

Hepatitis E virus (HEV) causes self-limited acute hepatitis in immunocompetent individuals and can establish chronic infection in solid organ transplant recipients taking immunosuppressive drugs. A well characterized small animal model is needed to understand HEV pathogenesis. In this study, we established a robust model to study acute and persistent HEV infection using Mongolian gerbils (*Meriones unguiculatus*) with or without immunosuppression. Gerbils were implanted subcutaneously with continuous release tacrolimus pellet to induce immunosuppression. Gerbils with or without tacrolimus treatment were inoculated with HEV intraperitoneally. Viremia, fecal virus shedding, serum antibody and ALT levels, liver histopathological lesions, hepatocyte apoptosis, and liver macrophage distribution were assessed. Mild to moderate self-limited hepatitis and IgM and IgG antibody responses against HEV ORF2 were observed in immunocompetent gerbils. Levels of HEV-specific IgM responses were higher and lasted longer in immunocompetent gerbils with higher peak viremia. Persistent viremia and fecal virus shedding with either weak, or absent HEV antibody levels were seen in immunosuppressed gerbils. Following HEV infection, serum ALT levels were increased, with lower and delayed peaks observed in immunosuppressed compared to immunocompetent gerbils. In immunocompetent gerbils, foci of apoptotic hepatocytes were detected that were distributed with inflammatory infiltrates containing CD68^+^ macrophages. However, these foci were absent in immunosuppressed gerbils. The immunosuppressed gerbils showed no inflammation with no increase in CD68^+^ macrophages despite high virus replication in liver. Our findings suggest adaptive immune responses are necessary for inducing hepatocyte apoptosis, CD68^+^ macrophage recruitment, and inflammatory cell infiltration in response to HEV infection. Our studies show that Mongolian gerbils provide a promising model to study pathogenesis during acute and persistent HEV infection.

## Author summary

Hepatitis due to genotype-3 E virus (HEV) is a zoonotic disease that is acquired by consumption of undercooked meat or offal from infected animals, typically pigs. HEV usually causes acute, self-limited hepatitis but in immunosuppressed persons, such as solid organ transplant recipients, it can establish chronic infection, associated with rapid liver fibrosis progression. Since HEV does not infect mice or other small animals, studies on disease pathogenesis are limited. In this study, we compared HEV kinetics, antibody responses, and liver disease in Mongolian gerbils with or without immunosuppression induced by the drug tacrolimus. In immunocompetent gerbils with acute infection, we observed mild hepatitis with foci of apoptotic hepatocytes and activated macrophages surrounding HEV-infected cells. In contrast, immunosuppressed gerbils failed to clear HEV infection and showed approximately 10-fold higher viral loads in serum and feces. Of note, despite higher virus replication in the liver, hepatocyte apoptosis and inflammation were not observed in the immunosuppressed group, suggesting that the liver damage in acute hepatitis E is immune-mediated. The absence of inflammation and apoptosis in livers of immunosuppressed gerbils with persistent, high-level HEV replication suggests that the progression to fibrosis in immunosuppressed patients is due to underlying factors other than HEV.

## Introduction

Hepatitis E virus (HEV) is an important cause of acute viral hepatitis in humans [1]. There are an estimated 20 million HEV infections worldwide, including 3.3 million symptomatic cases, and 44,000 HEV-associated deaths each year [1,2]. Most HEV infections have been reported as water-borne epidemic outbreaks in tropical and subtropical countries with poor sanitary infrastructure [3]. However, sporadic autochthonous HEV infections have been identified in developed countries of Europe, North America, and Asia, where HEV is mainly transmitted through eating raw or undercooked meat and liver [4]. Rare cases of blood transfusion transmitted HEV infections have also been reported worldwide [5]. In developed countries, most HEV infections are asymptomatic; however, cases of severe acute viral hepatitis and chronic HEV infections have been routinely reported in older and immunocompromised individuals, respectively [4].

Mammalian HEVs are classified under 3 genera, *Paslahepevirus*, *Rocahepevirus*, and *Chirohepevirus* [6]. Among them, *Paslahepevirus* also known as Orthohepevirus A (HEV-A) have been the causative agent for more than 99% of human infections reported worldwide, particularly genotypes 1–4 [7]. Recent reports suggest *Rocahepevirus ratti* also known as rat HEV is an emerging pathogen of acute viral hepatitis and chronic infection in humans in Hong Kong, Spain, France, and Canada [8–12]. However, in developed countries, HEV-A genotypes 3 and 4 are responsible for the majority of clinical cases of both acute and chronic HEV [4]. HEV-A genotypes 3 and 4 predominantly cause self-limited acute infections; however, a minority of these acute infections can lead to significant liver damage in the elderly which is attributed to effector memory CD8 T cell-mediated mechanisms [13]. Chronic HEV infections are almost exclusively observed in immunocompromised patients particularly in solid organ transplant (SOT) recipients but also in HIV infected individuals and patients with hematological malignancies [14]. Chronic HEV infection can lead to rapidly progressive liver disease, fibrosis, and sometimes cirrhosis in humans [14]. The immunocompromised status of an individual is the main risk factor for acquiring chronic HEV infection. Specifically, the immunosuppressive drug tacrolimus is currently used for the treatment and prevention of rejection in SOT recipients diagnosed with chronic HEV infection [15]. Tacrolimus (FK506) interacts with FK506 binding protein 12 (FKBP12) and inhibits calcineurin phosphatase activity in T cells [16] resulting in failure of NFAT-dependent cytokine gene expression which is necessary for complete T cell activation [17].

Animal models such as pigs, cynomolgus and rhesus monkeys, rabbits and immunodeficient human liver chimeric mice have been explored as models of acute HEV-A infection and immunocompromised models of chronic HEV-A infection [18–22]. The scope of rodent models, such as laboratory mice and rats, is highly limited for the study of HEV-A infection [23]. Rabbits and rats are small animal models currently available to study acute and chronic HEV infection, but they are only suitable to study rabbit variants of HEV-A genotype 3 and HEV-C1, respectively; human HEV-A genotypes 3 and 4 infections are limited in rabbits and absent in rats [20,24]. In contrast, Mongolian gerbils are a promising model to study acute infection with a broad range of HEV-A genotypes [25–29], as well as neurological manifestations of HEV-A infection [30,31]. Gerbils were also successfully infected with cDNA infectious clone-derived human HEV genotype 3 and 4 strains [32]. Furthermore, gerbils have the smallest body size among currently available HEV animal models making them cost-effective in studying chronic HEV infections with different immunosuppressive drug regimens.

In this study, we examined acute self-limited HEV-A genotype 3 infection in Mongolian gerbils and compared features of liver disease with those seen during persistent infection in gerbils treated with tacrolimus to induce immunosuppression. Importantly, hepatocellular apoptosis and macrophage recruitment were observed at sites of virus replication in livers of immunocompetent gerbils with acute HEV infection but were absent in immunosuppressed gerbils with high levels of intrahepatic HEV RNA replication. These data suggest that liver injury in acute HEV infection is immune-mediated and dependent upon responses targeted by tacrolimus.

## Results

### HEV infection in gerbils with or without tacrolimus treatment

**Fig 1:**
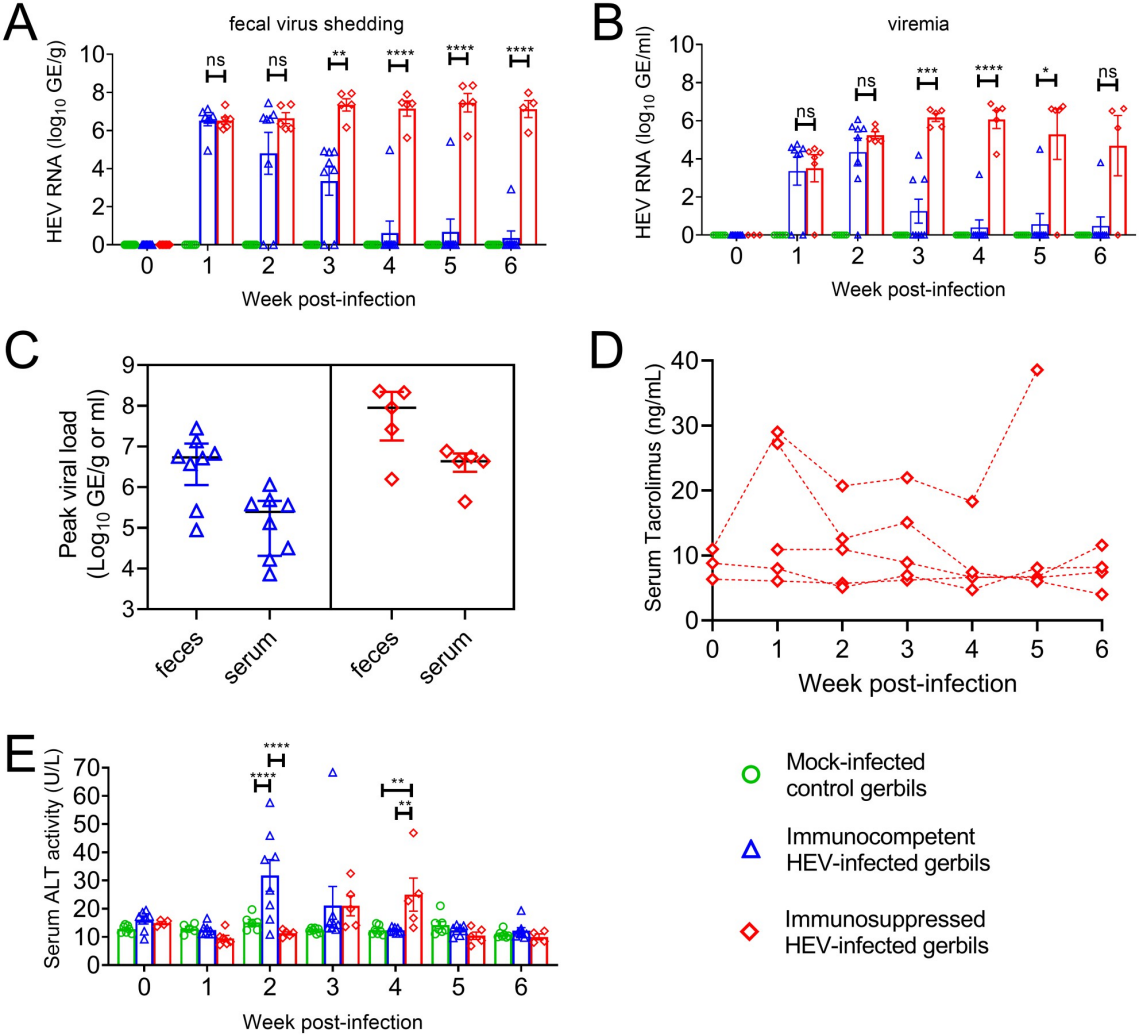
Acute and persistent infection with genotype 3 HEV in immunocompetent and immunosuppressed Mongolian gerbils. (A) Fecal virus shedding and (B) viremia measured in immunocompetent and immunosuppressed gerbils following intraperitoneal inoculation with the Kernow C1 isolate of genotype 3 HEV compared to mock-infected control gerbils. (C) Peak viral load measured in feces or serum from immunocompetent or immunosuppressed gerbils over 6 weeks. (D) Serum tacrolimus levels measured in immunosuppressed gerbils over 6 weeks. (E) Serum ALT levels measured in immunocompetent and immunosuppressed gerbils following HEV infection compared to mock-infected gerbils. Data are summarized as geometric means with standard deviations; symbols represent individual animals. Groups were compared by two-way ANOVA, Tukey’s multiple comparison test, **** p<0.0001, *** p<0.001, ** p<0.01, * p<0.05, n.s.: not significant.

To induce immunosuppression, gerbils were surgically implanted with controlled release tacrolimus pellets to reach levels similar to those observed in patients following SOT [33]. Untreated gerbils were defined as immunocompetent. Gerbils were infected with HEV-A genotype 3 at 2 weeks post-implantation when immunosuppression was established. The magnitude and duration of HEV infection was assessed by HEV RNA copies in feces and serum. All gerbils in the mock-infected control group were negative for HEV RNA in feces and serum throughout the experiment (Fig 1A and 1B). HEV RNA first appeared in feces and serum within one week after infection in both immunocompetent and immunosuppressed gerbils. In the first 2 weeks post-infection (wpi), mean viral loads were not significantly different in feces and serum of immunocompetent gerbils compared with those of immunosuppressed gerbils (p>0.05) (Fig 1A and 1B); however, significant differences in mean viral loads were observed between 3 to 6 wpi in feces and 3 to 5 wpi in serum (Two-way ANOVA, Tukey’s multiple comparison test, p<0.05) (Fig 1A and 1B). HEV RNA became undetectable in both feces and serum of 7 out of 8 immunocompetent gerbils at 4 wpi but persisted in the immunosuppressed gerbils until the end of the experiment (6 wpi) (Fig 1A and 1B). The means of the highest observed (peak) viral loads measured in feces and serum across 6 weeks were approximately 14-fold higher in immunosuppressed compared to immunocompetent gerbils (Fig 1C).

Tacrolimus concentration was measured in serum to assess the immunosuppression status of gerbils and was maintained at least at 6 ng/mL through the 6 weeks (Fig 1D). These levels were similar to those maintained in patients following SOT [33]. There was no apparent correlation between serum levels of tacrolimus and virus titers.

The mean serum ALT levels were significantly increased in immunocompetent gerbils at 2 wpi compared to mock-infected controls and returned to baseline levels by 3–4 wpi (Fig 1E). In immunosuppressed gerbils, a smaller increase in mean serum ALT levels was observed, which was also delayed compared to the immunocompetent group (Fig 1E).

### Antibody responses in gerbils infected with HEV

**Fig 2:**
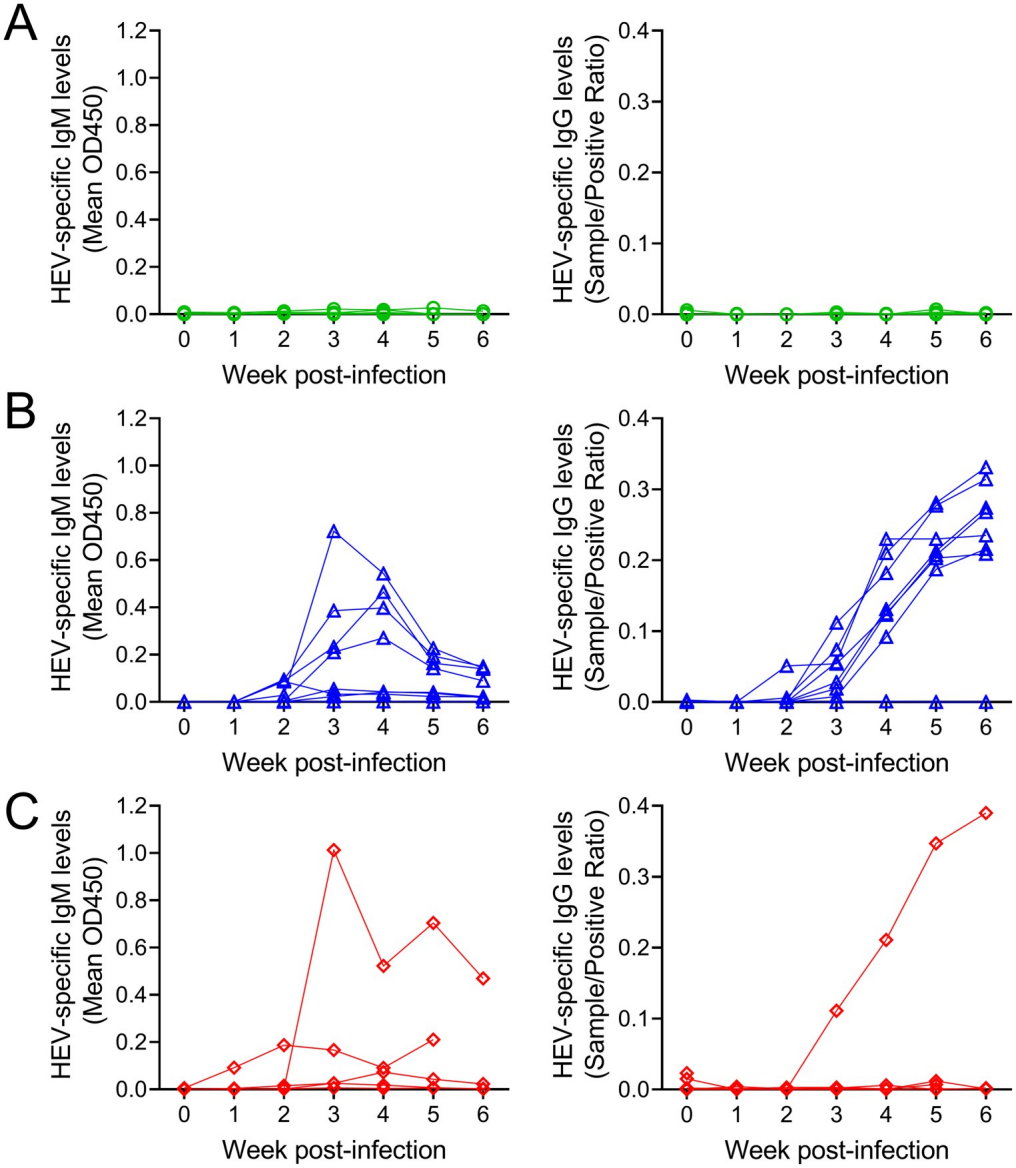
Antibody responses to HEV in immunocompetent and immunosuppressed gerbils compared to mock-infected controls. (A) Mock-infected control gerbils (n = 7), (B) Immunocompetent HEV-infected gerbils (n = 8), (C) Immunosuppressed HEV-infected gerbils (n = 5). Left: HEV-specific IgM. Right: HEV-specific IgG.

HEV ORF2-specific antibody responses in gerbil sera were measured by in-house indirect ELISAs. None of the mock-infected animals seroconverted to anti-HEV IgM or IgG positive (Fig 2A, left and right). Anti-HEV IgM was detected from 2 wpi in 4 of 8 immunocompetent gerbils; and the peak IgM was observed at 3 and 4 wpi (Fig 2B, left). In the immunosuppressed group, most gerbils showed weak or undetectable anti-HEV IgM responses (Fig 2C, left) with one outlier showing very high anti-HEV IgM from 3 wpi. Most immunocompetent gerbils (7 of 8) seroconverted to anti-HEV IgG positive by 4 wpi (Fig 2B, right). In contrast, most immunosuppressed gerbils (4 of 5) failed to seroconvert to anti-HEV IgG positive (Fig 2C, right). Collectively, HEV infection induced both IgM and IgG responses in immunocompetent gerbils while it induced weak IgM and no IgG responses in immunosuppressed gerbils.

### Kinetics of viremia and fecal virus shedding and correlation with serum antibody and ALT levels

**Fig 3:**
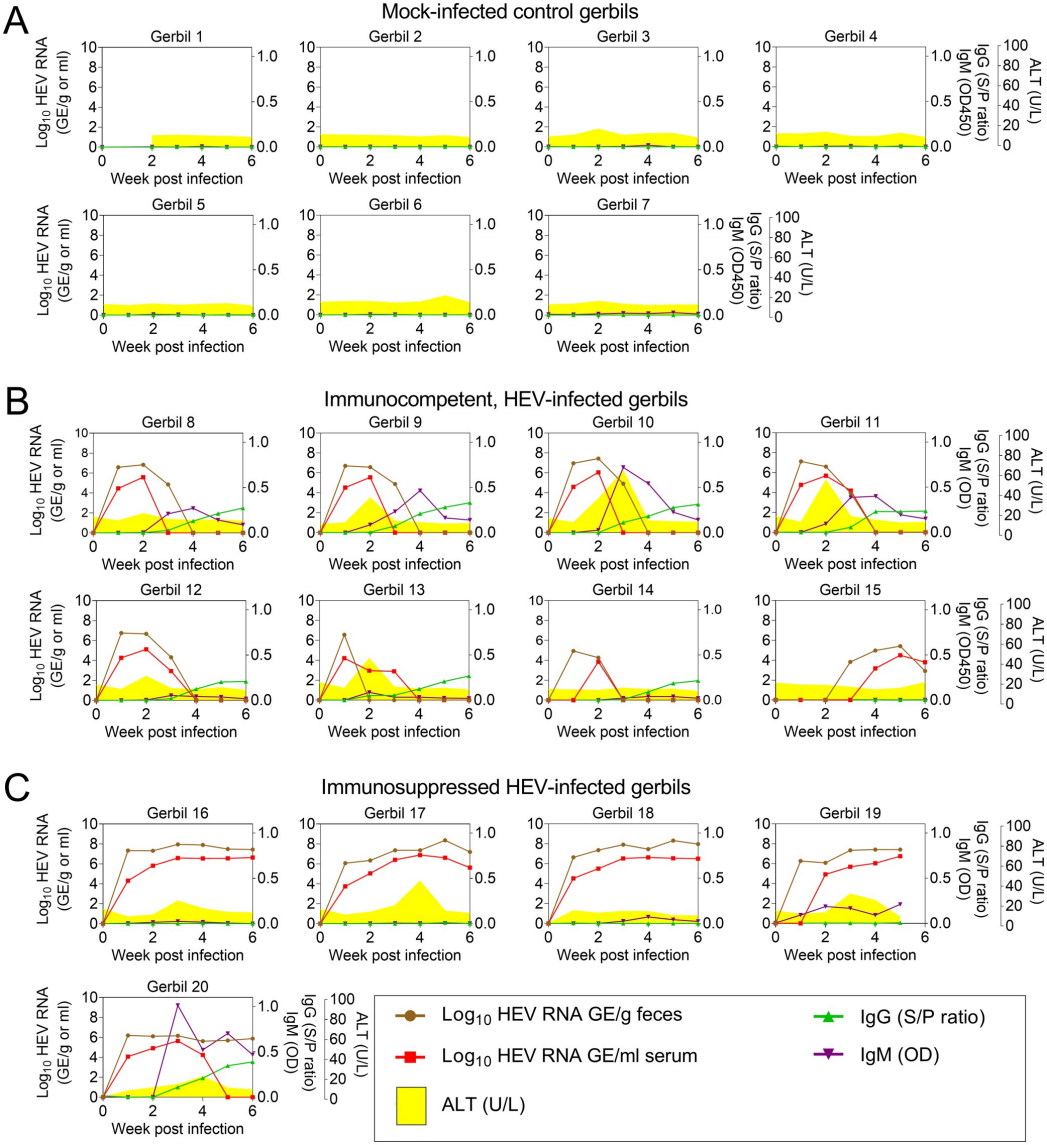
Biochemical, serologic, and virologic course of infection in immunocompetent gerbils compared to immunosuppressed gerbils. (A) mock-infected control Gerbils 1–7, (B) HEV-infected immunocompetent Gerbils 8–15, (C) HEV-infected immunosuppressed Gerbils 16–20. Each graph shows data from an individual gerbil. Viral load in feces and serum is plotted on the left y-axis in Genome Equivalents per g (feces) or ml (serum). Right hand y-axes show anti-HEV IgM levels (OD450), anti-HEV IgG levels (sample to positive ratio), and ALT levels (U/L). Sera and feces were collected weekly for 6 weeks (x-axis) from all gerbils except for Gerbil 19, which was euthanized at 5 wpi. Data shown in this figure for individual animals include viral load and antibody data that are also shown by treatment group in Figs 1 and 2.

Kinetics of viremia, fecal shedding, serum antibody responses, and ALT levels are displayed in Fig 3 for individual animals. Mock-infected control gerbils did not show viremia, fecal shedding, or elevated ALT (Fig 3A). None of the mock-infected animals seroconverted to anti-HEV IgM or IgG positive. Mean ALT in the mock-infected animals across all time points was 12.86 U/L (standard deviation 2.26 U/L).

**Fig 4:**
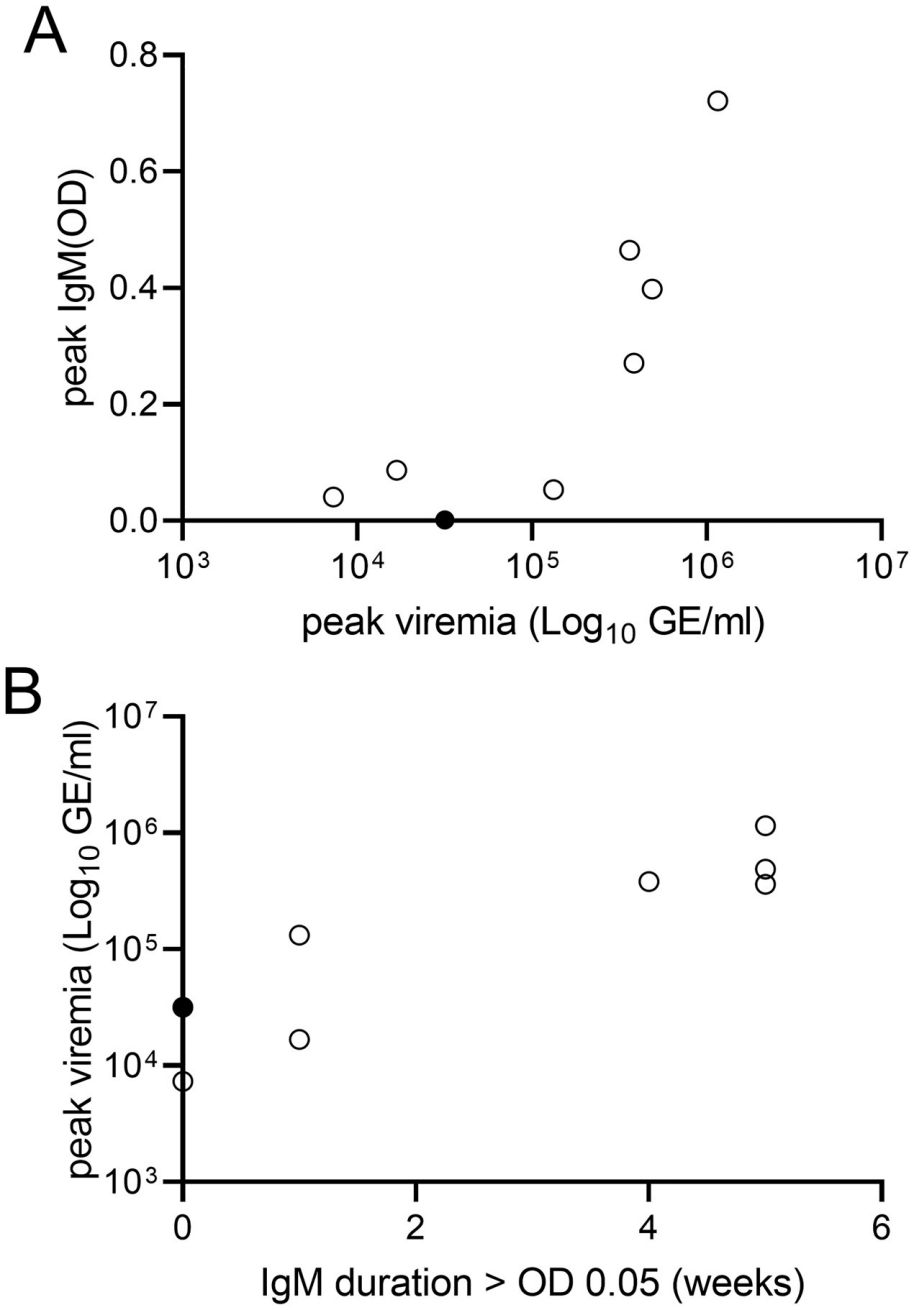
Peak viremia and IgM antibody responses in immunocompetent gerbils with acute HEV infection. (A) Relationship between peak viremia and peak levels of anti-HEV IgM in HEV-infected immunocompetent gerbils. The graph shows the peak viremia (log_10_ GE/ml) plotted against the peak anti-HEV IgM that was observed over 6 weeks. (B) Relationship between peak viremia and duration of detectable anti-HEV IgM (>0.05 O.D.) over 6 wpi. Peak viremia (log_10_ GE/ml) observed over 6 weeks is plotted against the number of weeks where anti-HEV IgM was detectable. In both graphs, each circle represents a single gerbil. By the end of the experiment (6 weeks), most gerbils had resolved their infection and viremia was undetectable in 7/8 gerbils (shown as open circles). Only one gerbil (filled circle) was still viremic at 6wpi. This gerbil showed delayed kinetics of viremia and had not seroconverted to anti-HEV IgM positive by the end of the experiment.

Following inoculation with HEV, immunocompetent gerbils showed similar kinetics of viremia and fecal shedding (Fig 3B, Gerbils 8–15) with one outlier (Gerbil 15, Fig 3B) in which the onset of fecal shedding and viremia were both delayed, and in which seroconversion did not occur by the end of the experiment. In gerbils with high viremia (peaks >3x10^5 copies per ml; Fig 3B, Gerbils 8–11), the magnitude and duration of the IgM response was greater than in gerbils with lower viremia (peaks <3x10^5 copies/ml; Fig 3B, Gerbils 12–14). This can be visualized by plotting peak viremia against either the peak of IgM (Fig 4A) or the duration of detectable IgM (Fig 4B). A Pearson’s correlation coefficient was computed to assess the linear relationship between peak viremia and peak IgM. There was a strong positive correlation between the two variables (r = 0.84, 95% confidence interval [CI] = 0.34–0.97, p = 0.009).

The ALT levels were elevated 2-4-fold in 5 of 8 immunocompetent gerbils. Peak ALT occurred at 2–3 weeks, around the same time that IgM begins to appear. Gerbils with higher anti-HEV IgM responses showed higher ALT values. Peak ALT values were strongly correlated with peak IgM levels (r = 0.79, 95% CI = 0.18–0.96, p = 0.021) and with peak viral load in feces (r = 0.78, 95% CI = 0.17–0.96, p = 0.023). Peak ALT values were not significantly correlated with peak viremia (r = 0.62, 95% confidence interval = -0.16–0.92, p = 0.104).

Immunosuppressed gerbils infected with HEV showed similar kinetics of viremia and fecal shedding (Fig 3C, Gerbils 16–19) except for Gerbil 20, which will be discussed separately. Gerbils 16–19 shed virus at higher levels in feces and serum compared to immunocompetent gerbils. Viral load increased rapidly, reaching a plateau by 2–3 wpi at >10^7^ copies/g in feces and >10^6^ copies/ml in serum. Anti-HEV IgM and IgG were undetectable in 2 of 5 gerbils (Fig 3C; Gerbils 16 and 17). Low levels of anti-HEV IgM were detected in 2 of 4 animals with no seroconversion to anti-HEV IgG positive (Gerbils 18 and 19).

In Gerbil 20, a strong IgM response was detected at 3 wpi accompanied by the appearance of anti-HEV IgG. In this gerbil, viral loads were 1–2 log lower in serum and feces compared to other immunosuppressed gerbils; and HEV RNA became undetectable in serum by 5 wpi but remained detectable in feces throughout the experiment (6 wpi). Serum tacrolimus levels in this gerbil were not lower than in other gerbils at weeks 1–4 but they showed a declining trend over time such that by 6 wpi they had fallen to 4 ng/ml.

Elevated ALT levels were observed in most immunosuppressed gerbils except Gerbil 18, but the ALT peak was lower and occurred later compared to the immunocompetent animals (Fig 3C). The mean time until the ALT peak was significantly shorter (p<0.01) in the immunocompetent group (15.2 days) compared to the immunosuppressed group (24.5 days). Importantly, in immunosuppressed HEV-infected gerbils, there was no relationship between the ALT peak and either viremia, fecal shedding, or antibody responses.

### Tissue viral load and histopathology in HEV-infected gerbils

**Fig 5:**
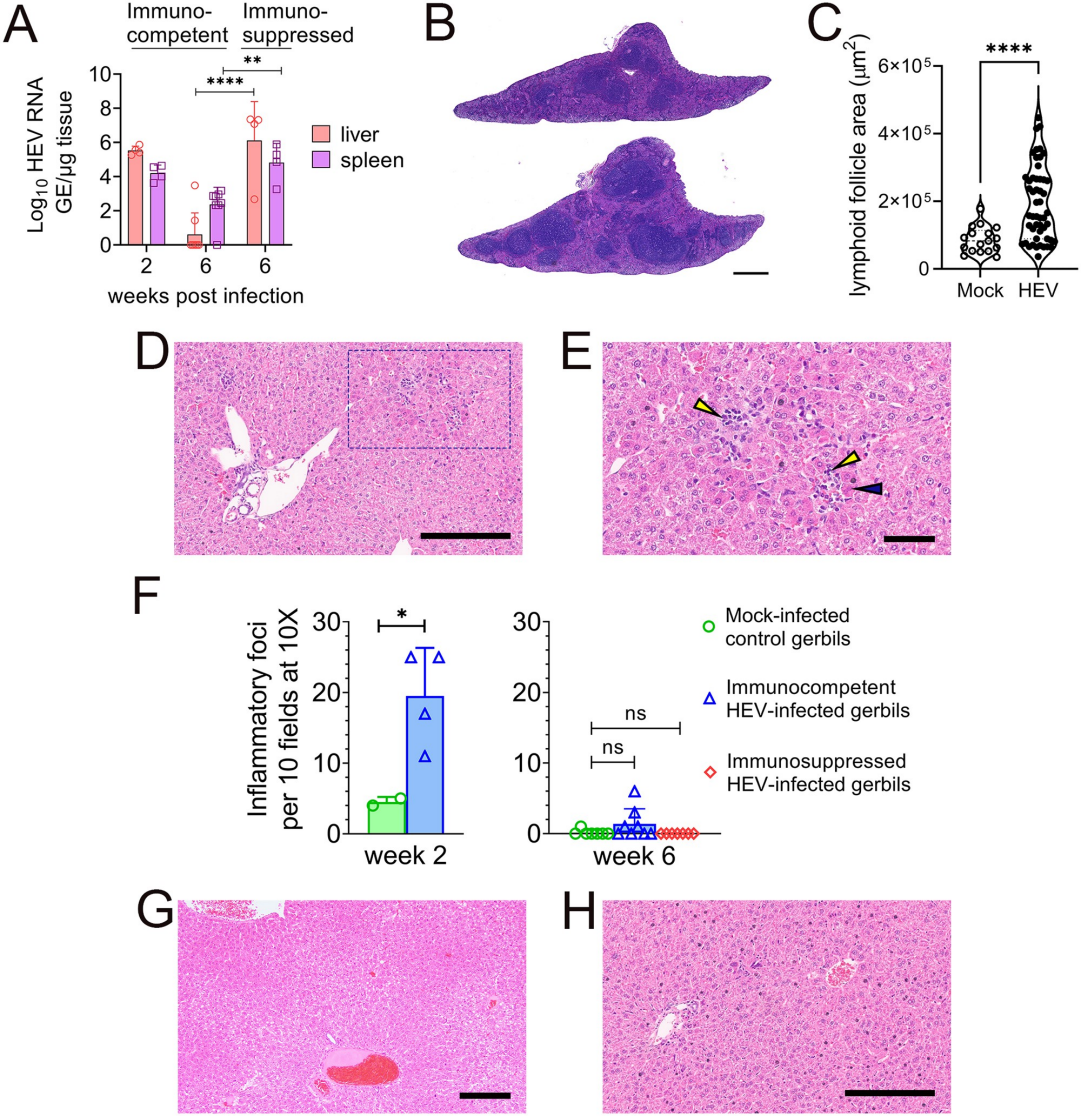
Tissue viral load and histopathological lesions in liver. (A) HEV RNA levels in liver and spleen from HEV-infected immunocompetent gerbils at 2 wpi (peak viremia), and 6wpi (convalescent phase) and in HEV-infected immunosuppressed gerbils at 6 wpi (persistent infection). Groups were compared by Welch’s t test. ** indicates p<0.01. (B) Hematoxylin and eosin (H&E) staining of FFPE spleen tissue from a mock-infected control gerbil (top) and an HEV-infected immunocompetent gerbil (bottom) at 2 wpi. Scale bar: 500 μm. (C) Area of lymphoid follicles in spleen tissue sections from HEV-infected immunocompetent gerbils (n = 4) compared to mock-infected control gerbils (n = 2). Groups were compared by Mann Whitney test **** indicates p<0.0001. (D) Hematoxylin and eosin- (H&E-) stained liver sections from an HEV-infected immunocompetent gerbil at peak viremia (2wpi), Scale bar 200 μm and (E) at higher magnification to show foci of lymphocytes (yellow arrowheads) surrounding hepatocytes with hypereosinophilic cytoplasm (blue arrowhead) that were sporadically apoptotic. Scale bar 100 μm. (F) Quantification of inflammatory foci in livers of mock-infected or immunocompetent and immunosuppressed gerbils at 2 or 6 weeks following HEV infection. Foci were enumerated by a veterinary pathologist blinded to the animal groups. Groups were compared by Welch’s t test (one-tailed) * indicates p<0.05, ns indicates not significant. (G) H&E-stained liver sections from an immunosuppressed gerbil with persistent HEV infection at 6wpi. Scale bar 200μm. (H) H&E-stained liver sections from a mock-infected control gerbil, Scale bar 200μm.

HEV RNA was detected at high levels in liver and spleen tissues of all immunosuppressed gerbils at 6 wpi while they were below detectable levels in liver tissues of most immunocompetent gerbils in the convalescent phase at 6 wpi (Fig 5A). HEV RNA was still detected at significant levels in the spleen of immunocompetent gerbils for up to 6 wpi but at lower levels compared to HEV-infected immunosuppressed gerbils (Fig 5A).

Macroscopic examination identified no differences between livers of HEV-infected gerbils (either immunocompetent or immunosuppressed) and control animals. However, HEV-infected immunocompetent gerbils showed larger spleens (Fig 5B) with enlarged lymphoid follicles compared to mock-infected controls (Fig 5C).

Histopathological evaluation of H & E-stained liver sections showed mild hepatitis in immunocompetent gerbils at peak viremia (2 wpi) which was characterized by multiple foci of lymphocytes surrounding hepatocytes with hypereosinophilic cytoplasm that were sporadically apoptotic. These foci were randomly scattered within the parenchyma (Fig 5D and 5E). Foci of inflammation were enumerated by a veterinary pathologist blinded to the animal groups. The mean number of foci was 1.95 per 10X field in livers of immunocompetent gerbils at 2 wpi (Fig 5F). The necroinflammation was mostly resolved at 6 wpi during the convalescent phase (Fig 5F). In contrast, livers of immunosuppressed gerbils were nearly normal at 6 wpi (Fig 5G) with no evidence of necroinflammation despite persistent and higher levels of HEV replication in hepatocytes. Livers of mock-infected gerbils were mostly normal (Fig 5H) with rare inflammatory cell foci, but these were characterized by a greater proportion of neutrophils than the lymphocyte-rich foci observed in infected gerbils. Occasional foci of periportal extramedullary hematopoiesis were found in all groups of gerbils, but there were no significant inflammatory foci involving the portal tracts. In summary, HEV induced mild hepatitis that correlated with peak virus replication in the immunocompetent gerbils while it did not induce necroinflammation in immunosuppressed gerbils despite persistent 10-fold higher levels of replication in liver.

### Focal distribution of HEV ORF2 antigen in liver sections of gerbils

**Fig 6:**
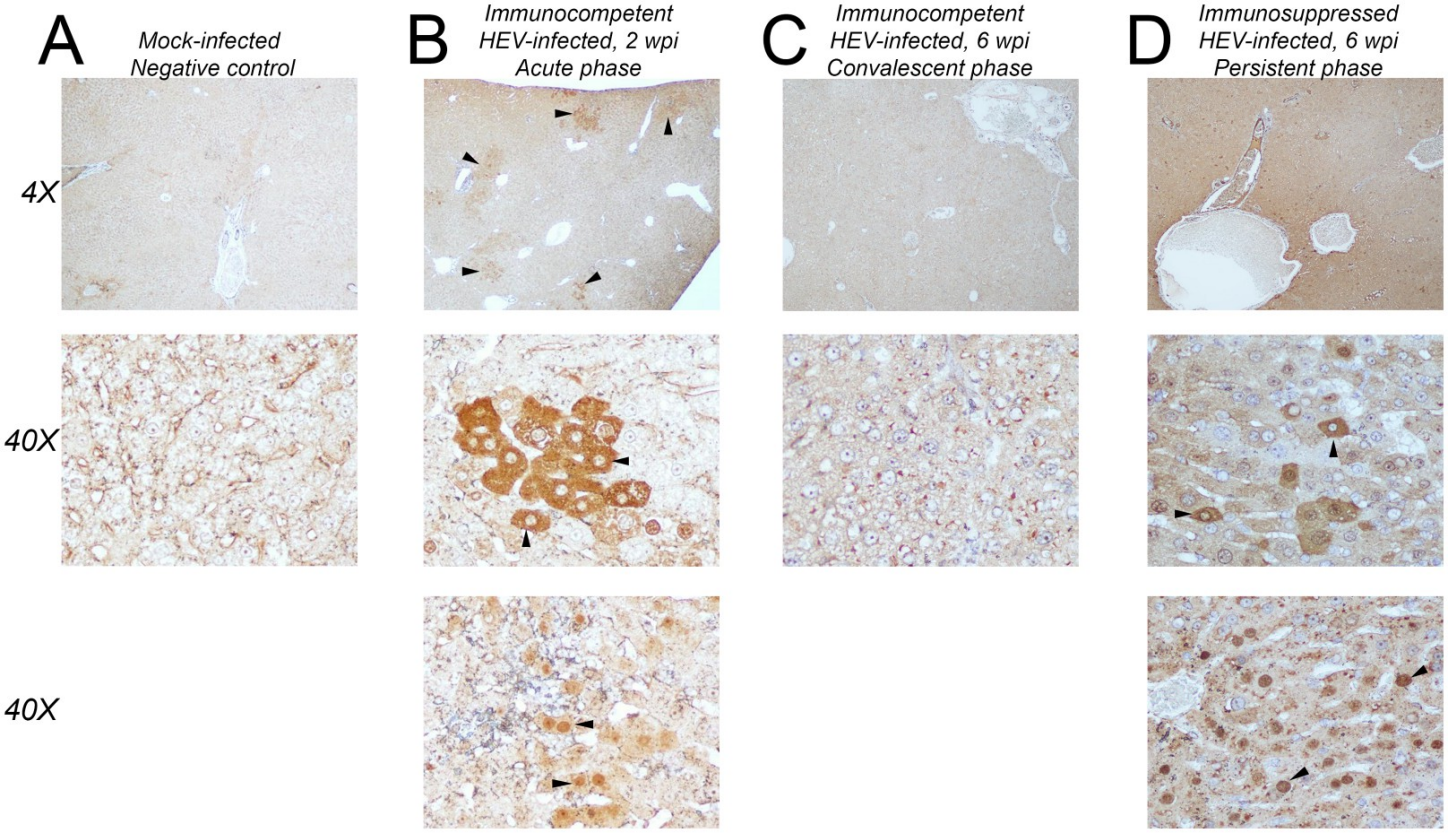
Immunohistochemistry detection of ORF2 antigen in FFPE liver sections. (A) Mock-infected control gerbil at 2 wpi. (B) HEV-infected immunocompetent gerbil at 2 wpi (peak viremia). Black arrowheads show examples of clusters of ORF2-positive hepatocytes at 4X magnification or individuals ORF2-positive hepatocytes at 40X magnification. (C) HEV-infected immunocompetent gerbil at 6 wpi (convalescent phase). (D) HEV-infected immunosuppressed gerbil at 6 wpi (persistent infection). Most hepatocytes were positive for ORF2 with more intense staining observed in a subset of hepatocytes. Black arrowheads show examples of individuals ORF2-positive hepatocytes at 40X magnification. Low magnification images (4X) and high magnification images (40X) are shown. For the immunocompetent, HEV-infected gerbils at 2 wpi (B) and the immunosuppressed HEV-infected gerbils at 6 wpi (D), representative high magnification images of HEV-infected hepatocytes are shown with either predominantly cytoplasmic (middle panels) or predominantly nuclear (bottom panels) ORF2.

The distribution and intracellular localization of HEV ORF2 antigen was assessed in liver sections by immunohistochemical staining with anti-ORF2 antibodies. ORF2 antigen staining was not detected in negative control liver sections (Fig 6A). Localized clusters of ORF2-positive cells were detected in the liver parenchyma, specifically in periportal and lobular areas in immunocompetent gerbils at 2 wpi (Fig 6B). The immunocompetent gerbils in the convalescent phase of HEV infection (6 wpi) did not show any apparent ORF2 antigen staining in the liver sections (Fig 6C). In contrast, a diffuse pattern of ORF2 antigen staining with occasional clustering of intensely stained cells was observed throughout the liver parenchyma in immunosuppressed gerbils at 6 wpi (Fig 6D). In both immunocompetent (Fig 6B) and immunosuppressed gerbils (Fig 6D) infected with HEV, ORF2 antigen was predominantly detected in the cytoplasm of most infected hepatocytes. However, there were also some foci of infected hepatocytes where ORF2 was predominantly found in the nuclei (Fig 6B and 6D, bottom panels).

### Recruitment of CD68^+^ macrophages and hepatocyte apoptosis during acute HEV infection of gerbils

**Fig 7:**
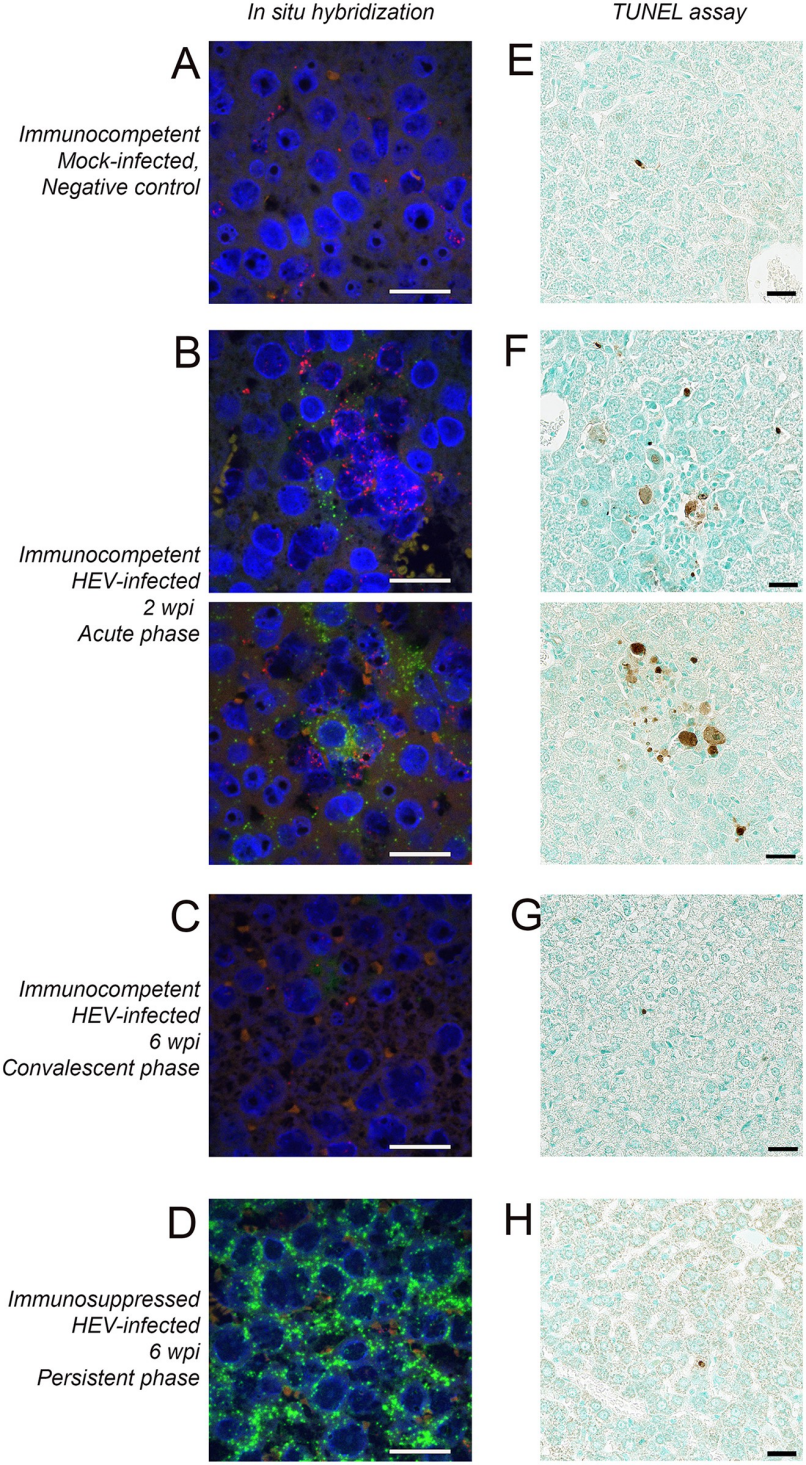
Visualization of HEV genome RNA, CD68 mRNA positive cells and apoptotic cells in FFPE liver sections. Panels A-D show representative images of FFPE liver sections stained by fluorescent in situ hybridization to detect HEV positive sense RNA (green) and gerbil CD68 mRNA (red). Sections were counterstained with DAPI to visualize nuclear DNA. Panels E-H show representative images of FFPE liver sections stained by TUNEL assay to detect apoptotic cells. Sections were counterstained with methyl green. (A and E) mock-infected control at 2wpi, (B and F) HEV-infected immunocompetent gerbil at 2 wpi (peak viremia; two representative images), (C and G) HEV-infected immunocompetent gerbil at 6 wpi (convalescent phase), (D and H) HEV-infected immunosuppressed gerbil at 6 wpi (persistent infection). Scale bar represents 25μm in all images.

HEV genomic RNA and CD68 mRNA were visualized in gerbil liver sections by fluorescent in situ hybridization. HEV RNA was not detected in livers of mock-infected control gerbils (Fig 7A). In HEV-infected immunocompetent gerbils at 2 wpi (peak viremia), HEV RNA was detected in the cytoplasm of hepatocytes. HEV RNA-positive hepatocytes showed a focal distribution, similar to the distribution of ORF2 in livers of immunocompetent gerbils at peak viremia. In immunocompetent gerbils at 2 wpi, CD68^+^ macrophages were often observed adjacent to or surrounding HEV RNA-positive hepatocytes (Fig 7B). By 6 wpi (convalescent phase), HEV RNA was absent from the hepatocytes and there were no CD68^+^ macrophages (Fig 7C). In immunosuppressed gerbils with persistent HEV infection, HEV RNA was detected at higher levels, and in almost every hepatocyte (Fig 7D), but CD68^+^ macrophages were absent.

The terminal deoxynucleotidyl transferase dUTP nick end labeling (TUNEL) assay was used to detect apoptotic cells in liver sections from gerbils infected with HEV. In livers of mock-infected gerbils, most cells were TUNEL-negative with rare, individual apoptotic cells observed in a background of non-apoptotic cells (Fig 7E). In livers of HEV-infected immunocompetent gerbils at peak viremia, foci of apoptotic cells (mostly hepatocytes but also some immune cells) were observed scattered in the parenchyma (Fig 7F). By 6 wpi (convalescent phase), the foci of apoptotic cells were cleared from the liver and levels of apoptotic cells were similar to mock-infected controls (Fig 7G). In immunosuppressed gerbils with persistent HEV infection, levels of apoptosis in the liver appeared indistinguishable from the mock-infected control gerbils (Fig 7H). These data indicate that apoptosis and recruitment of CD68^+^ macrophages to HEV-infected hepatocytes are adaptive immune-mediated processes that are suppressed by tacrolimus treatment.

## Discussion

Although progress has been made in the development of animal models for the study of HEV infection and disease pathogenesis [23], none of the current models fully reproduce the disease seen in humans. HEV infection may be acute or chronic depending upon the host immune status. However, the histopathological features of acute and, especially, chronic hepatitis E remain poorly defined. Whether the liver damage is preexisting to HEV infection, caused by HEV, or unrelated and sustained by other causes, remains to be fully elucidated. Moreover, the degree of liver damage among patients with chronic HEV infection is highly variable, ranging from near-normal liver to acute hepatitis, or mild to moderate chronic hepatitis. In the largest published study, variability in the histopathology of hepatitis E correlated with patient immune status and pre-existing liver disease, highlighting the critical role played by these two factors in determining the variability and severity of liver disease associated with persistent HEV infection [34].

In this study, taking advantage of the promising gerbil model of HEV infection [26,27], we characterized the virological, clinical, and histopathological features of acute and chronic HEV infection in immunocompetent and immunosuppressed gerbils. We have shown that tacrolimus is sufficient to establish persistent HEV infection in gerbils, as reported in cynomolgus monkeys [18], and in line with previous findings that tacrolimus intake is a reliable predictive factor of chronic HEV infection in immunosuppressed patients [15]. Our data in gerbils recapitulate key features of hepatitis E in humans. Mild hepatitis with increased hepatocyte apoptosis and activated macrophages surrounding HEV-infected cells was observed in the immunocompetent gerbils at peak viremia. In contrast, hepatocyte apoptosis and liver inflammation were not seen in tacrolimus-treated immunosuppressed animals with persistent HEV infection and high levels of intrahepatic HEV RNA, suggesting that the liver damage is immune mediated.

The gerbil model provides detailed information on the kinetics of serum biomarkers relevant for diagnosis. In immunocompetent gerbils with acute HEV infection, viremia is transient and detectable for only 1–3 wpi. The mean time to the ALT peak is 2–3 weeks. Thus, by the time an increase in ALT is observed, viremia may be declining or undetectable. In contrast, IgM persisted longer, underscoring the importance of anti-HEV IgM as the first line marker for diagnosis of acute HEV infection. There was a clear correlation between the magnitude of anti-HEV IgM response and the peak viral load measured in serum and feces (Figs 3B and 4A). The strong correlation of serum IgM levels with peak viremia suggests HEV replication levels determine the magnitude of the anti-HEV IgM antibody response in gerbils. Although data on viral HEV kinetics are very limited in patients, it is likely that the extent of HEV replication modulates the antibody response in humans. Peak ALT was also strongly correlated with the peak viral load in feces. Together, these observations suggest that anti-HEV IgM antibody levels may be higher in clinically apparent cases of acute hepatitis E with elevated ALT levels compared to subclinical cases such as asymptomatic blood donors with viremia.

The gerbil model recapitulates the kinetics of the antibody response to HEV seen in humans with acute and chronic infection. In immunocompetent gerbils, anti-HEV IgG become detectable within one week after the appearance of anti-HEV IgM. The levels of anti-HEV IgG continued to increase over time until the end of the experiment (6wpi) in the immunocompetent gerbils. In contrast, most of the HEV-infected immunosuppressed gerbils did not seroconvert to anti-HEV IgM or IgG positive, consistent with reports of poor HEV antibody detection in chronic HEV-infected immunosuppressed patients [35]. Rarely, chronic HEV-infected patients develop spontaneous HEV antibody responses and subsequently clear the infection [36]. Consistent with these findings, a strong HEV antibody response was observed in only 1 of 5 immunosuppressed gerbils (Gerbil 20). In this animal, the antibody response was followed by clearance of viremia and a small reduction of fecal virus shedding. Thus, it is likely that in this gerbil, which showed decreasing levels of serum tacrolimus as HEV infection progressed, the immunosuppression was inadequate to suppress B-cell immune responses but sufficient to allow persistent virus replication in the liver. These data suggest that the antibody response and the viral kinetics of HEV in immunosuppressed patients was largely determined by the serum concentration of tacrolimus treatment after SOT.

The distribution of HEV ORF2-positive hepatocytes in the livers of infected gerbils, closely resembles the pattern seen in biopsy specimens from patients with acute and chronic HEV infection [37]. Furthermore, in HEV-infected gerbil hepatocytes ORF2 was either predominantly cytoplasmic, predominantly nuclear, or distributed in both the cytoplasm and the nucleus, as seen previously in humans [37]. Thus, these data support a role for nucleocytoplasmic shuttling of ORF2 during the viral life cycle.

In liver sections from gerbils with acute HEV infection at 2 wpi, the apoptotic cells, ORF2-positive hepatocytes, and inflammatory infiltrates all occurred in clusters or foci distributed through the parenchyma. The clusters or foci of apoptotic cells observed in our study are in contrast with findings in an earlier study of genotype 4 HEV infection in gerbils [27] where a majority of hepatocytes were apoptotic by 2–3 wpi. Surprisingly, however, such extensive hepatocellular apoptosis was not associated with severe liver disease. In our study, the foci of apoptotic hepatocytes and immune cells in a background of non-apoptotic hepatocytes is consistent with the mild hepatitis and the extent of ALT elevation observed in immunocompetent gerbils with acute HEV infection. The role of different HEV genotypes in inducing hepatocellular apoptosis and liver damage remains to be established.

The spleen is the major site of systemic immune response against HEV with enlarged lymphoid follicles observed in the immunocompetent gerbils. In immunocompetent gerbils, significant residual HEV RNA was present in the spleen at 6 wpi even after the resolution of hepatitis when viremia was undetectable. Accumulation of viral RNA in the spleen has been observed in HEV-infected gerbils [29] and in mouse models of hepatitis A virus [38] and likely reflects sequestration of virus by phagocytic cells.

Infection with HEV genotype 3 in humans is typically characterized by both lobular and portal inflammation with scattered apoptotic hepatocytes and sometimes focally clustered activated Kupffer cells or macrophages indicating piecemeal necrosis [39]. In our study, the histopathological features of mild hepatitis seen in the liver of immunocompetent gerbils were similar to those observed in HEV genotype 3 infected immunocompetent individuals [39]. In contrast to immunocompetent gerbils, those immunosuppressed showed no apparent signs of liver inflammation. These data are in line with those reported in humans, where the degree of liver damage has been shown to be highly variable and depending on the immunologic status and pre-existing liver condition [36], with a trend characterized by little activity in immunocompromised patients [36]. The inapparent liver inflammation that we have seen in persistently infected gerbils is similar to that observed in SOT recipients during the early phase of chronic HEV infection [40]. Our data show that under immunosuppressive conditions, hepatocyte apoptosis is absent in HEV-infected gerbils. In contrast, multiple foci of apoptotic hepatocytes were observed in liver tissues of immunocompetent gerbils associated with inflammatory foci suggesting that immunological mechanisms underlie hepatocyte killing and initiation of inflammation in HEV-infected liver tissues. It is likely that hepatocyte apoptosis is intricately connected to hepatic inflammation and development of adaptive immune responses against HEV as seen in other forms of viral hepatitis [41].

CD68 is expressed in Kupffer cells and monocyte derived macrophages in liver, both of which can promote inflammation. CD68^+^ macrophages are diffusely located in portal and lobular areas in healthy liver tissues but are increased in the portal areas of diseased liver tissues as commonly seen in chronic viral hepatitis [42,43]. In our study, the presence of clusters of CD68^+^ macrophages in the vicinity of HEV-infected cells suggests that liver macrophages may play an important role in killing and clearing HEV-infected hepatocytes as well as promoting inflammation in livers of immunocompetent gerbils. In contrast, CD68^+^ macrophages were reduced in livers of immunosuppressed gerbils and not colocalized with HEV-infected cells.

Previous studies showed that a human strain of HEV genotype 3 replicated persistently in cyclosporin treated rabbits albeit at low levels and induced liver fibrosis in about 25–50% of animals at 8 and 13 wpi [20]. However, in tacrolimus treated gerbils, we did not see fibrosis in liver parenchyma at 6 wpi, as assessed by Masson’s Trichrome staining. This may be explained by the absence of significant hepatocyte apoptosis and inflammation, which would be required for hepatic stellate cell activation and fibrogenesis [44,45]. Also, we did not follow persistent infection past 6 weeks as the tacrolimus pellets were designed for continuous release only up to that timepoint. Furthermore, the causal role of HEV in inducing fibrosis is not clearly established in SOT patients diagnosed with chronic HEV and the progression to fibrosis in these patients is infrequent which may be due to causes other than HEV such as chronic allograft dysfunction [46] or other causes as recently suggested [34].

Slow-release pellets implanted subcutaneously were chosen to deliver tacrolimus to the gerbils to achieve a more reliable dosing compared to oral delivery (in water or food) where the amount of tacrolimus consumed per animal per day may be variable. A potential limitation of the subcutaneous implantation approach to deliver tacrolimus is that the immunosuppression is continuous, which might have resulted in weight loss in some gerbils.

In summary, our study demonstrates that Mongolian gerbils are highly susceptible to infection with human HEV genotype 3 strain, showing typical features of acute viral hepatitis that resemble those seen in humans, with foci of apoptotic hepatocytes and activated macrophages surrounding HEV-infected cells. As observed in SOT recipients [15], tacrolimus treatment induced persistent HEV infection in gerbils. However, inflammation and hepatocyte apoptosis were absent in the tacrolimus-treated gerbils with persistent HEV infection. These data suggest that liver damage during acute HEV infection is immune-mediated. Although chronic HEV infection has been associated with rapid fibrosis progression in some patients, there is little information on the underlying mechanisms. In our study, the absence of inflammation and apoptosis in immunosuppressed gerbils with persistent HEV infection suggests that the progression to fibrosis in immunosuppressed patients (e.g. SOT recipients) is due to underlying factors other than HEV.

## Methods

### Ethics statements

No human subjects were involved in this study. All animal procedures were approved by the Food and Drug Administration Institutional Animal Care and Use Committee (protocol 2019–12).

### Animal experiments

Two animal experiments were conducted to examine acute and chronic HEV infection and pathogenesis at 2 wpi and at 6 wpi. Mongolian gerbils were purchased from Charles River Laboratories. Inoculum containing HEV was freshly prepared for each experiment from a 20% fecal suspension derived from a Rhesus macaque that had been experimentally infected with the genotype 3 HEV strain Kernow C1 (provided by Professor Christopher Walker from the Research Institute at Nationwide Children’s Hospital). The Kernow C1 strain was originally isolated from the stool of an HIV-positive patient with chronic hepatitis E [47]. To prepare the inoculum for gerbil experiments, the macaque-derived fecal suspension was subjected to two rounds of chloroform extraction and 1-octanol extraction followed by ultracentrifugation at 110,000 x g. The final pellet was resuspended in PBS and sterile filtered.

Experiment 1: Six gerbils (16-week-old males) were divided into 2 treatment groups, control (n = 2) and HEV-infected group (n = 4). After 7-day acclimatization period, all gerbils in the HEV-infected group were infected with HEV at 1.6 x 10^7^ GE/animal intraperitoneally. Animals in the control group received PBS. Fecal and serum samples were collected weekly. All animals were humanely euthanized at 2 wpi and tissues were harvested.

Experiment 2: Based on the data from a small pilot study (RNAemia, fecal virus RNA, serum ALT data, and liver pathology scores), we performed a statistical power analysis to predict the minimum gerbil sample size per treatment group which was found to be 5 (p = 0.05 and 80% power). Since no prior viremia and fecal virus data were available for immunosuppressed gerbils, we assumed a 1 log increase in HEV viral load in the immunosuppressed gerbils would represent a meaningful difference. We also considered a 0.1 log increase in SD for the immunosuppressed gerbil group over the SD of the immunocompetent gerbils obtained in the pilot study. We included 3 additional gerbils in each treatment group based on expectation that up to 3 animals in the tacrolimus treated group would be removed from the study as they may reach the humane end points of the study due to the general side effects of immunosuppression.

A total of twenty-four gerbils (24-week-old males), were divided into 3 treatment groups, the control group (n = 8), the immunocompetent HEV-infected group (n = 8), and the immunosuppressed HEV-infected group (n = 8). After a 7-day acclimatization period, gerbils in the immunosuppressed group were surgically implanted with a single controlled release tacrolimus pellet (Innovative Research of America, 25 mg tacrolimus per pellet) subcutaneously in the neck region. The tacrolimus dose for gerbils was calculated by extrapolating from the corresponding dose used in a rat model of immunosuppression with subcutaneous implantation of tacrolimus pellets [48] by interspecies allometric scaling [49]. For the dose conversion, gerbil body surface area was calculated as 10.5x(weight in grams)^2/3^ [50]. Surgical wound clips were removed after 10 days. Two weeks after tacrolimus pellet implantation, gerbils in the two treatment groups were infected with HEV at 1 x 10^7^ GE/ animal intraperitoneally. Animals in the control group received PBS. Fecal and serum samples were collected weekly. In the immunosuppressed HEV-infected group, 3 and 1 animals were removed from the study in the 3^rd^ and 7^th^ week after tacrolimus pellet implantation, respectively. In the control group, 1 animal was removed from the study in the 4^th^ week. These animals experienced rapid weight loss and/or reached humane end points prompting their removal from the study by euthanasia. Data from the immunosuppressed HEV-infected animal that was euthanized at 7 weeks after tacrolimus implantation (5 wpi) were included in the analyses except for the missing time point at 6 wpi. All remaining animals were humanely euthanized at 6 wpi and tissues were harvested.

### Quantification of HEV RNAs in feces, serum and tissue samples

Serum samples (25–50μl) were diluted in PBS and 20% fecal suspensions were prepared in PBS and RNA was isolated using the QiaAmp Viral RNA Mini Kit (Qiagen) according to the manufacturer’s instructions. RNA was isolated from tissue samples using the RNeasy Mini Kit (Qiagen) according to the manufacturer’s instructions with on-column DNase digestion to remove genomic DNA. Quantification of HEV RNA used a method based on previously reported protocols [51,52] with modifications in primer and probe designs [53]. The limit of detection of this assay is approximately 15 RNA copies per reaction. Standard curve was calculated using 10-fold serial dilutions of purified *in vitro* transcribed, infectious clone-derived full-length HEV RNAs (Kernow C1, p6).

### HEV IgM and IgG ELISA

A truncated ORF2 gene expressing amino acids 422–637 was cloned into the pET28a(+) (EMD Biosciences) expression vector with a C-terminal His tag. The resulting plasmid was used to transform *E*.*coli* BL21(DE3) cells (Thermo Fisher Scientific), which were grown in LB medium at 37°C to an optical density at 600 nm of 0.6–0.7. Expression of the His-tagged protein was induced by addition of IPTG (isopropyl-β-d-thiogalactopyranoside) to a final concentration of 0.5 mM with further culture for 2 h. The cells were subsequently pelleted by centrifugation and the pellet resuspended in binding buffer (50 mM NaH_2_PO_4_, pH 8.0, 300 mM NaCl, and 10 mM imidazole) supplemented with cOmplete mini protease inhibitor (1 tablet for 10 ml buffer), Lysozyme (1mg/ml) and Benzonase nuclease (25U/ml). The cell suspension was incubated on ice for 30 min and homogenized in a homogenizer. Cell lysates were clarified by centrifugation 14000 x g for 30 min at 4°C. His-tagged truncated ORF2 was purified by affinity chromatography using HisPur Ni-NTA superflow agarose (Thermo Fisher Scientific) in 1 ml polypropylene columns (Qiagen). Maxisorp ELISA plates (Thermofisher Scientific) were coated with 100 μl per well of recombinant HEV ORF2 antigen (2 μg/ml) in ELISA coating buffer (Biolegend) overnight at 4°C. After 3 PBS washings, plates were blocked with 150 μl of 5% non-fat milk prepared in PBS for 2h at 37°C. Serum samples were diluted 1:100 in 5% non-fat milk prepared in PBS-Tween20 (0.05%) and 100 μl was added into each well. 5% non-fat milk only served as negative control. The duplicate wells were prepared for each sample and control. The plates were incubated for 1h at 37°C. The plates were washed 5 times with PBS-Tween20 (0.05%). The plates were incubated with 100 μl of HRP-conjugated anti-mouse IgM (Thermofisher Scientific) or anti-mouse IgG (Sigma Aldrich) per well for 1h at 37°C. Secondary antibodies were diluted in 5% non-fat milk at 1:1000 (anti-IgM) or 1:20000 (anti-IgG). The plates were washed 5 times with PBS-Tween20 (0.05%). The plates were incubated with 100 μl per well of SureBlue TMB microwell peroxidase substrate (KPL) for 5 min at RT in dark. Reaction was stopped with 100 μl of 1N HCl per well. Absorbance was measured at 450 nm in a microplate reader (Biotek Synergy neo2). The OD450 values of test samples and positive controls were subtracted from those of negative control. The final OD450 value of each sample was obtained by calculating the average of values from duplicate wells. For IgG, sample to positive ratio (S/P) ratio was calculated by dividing the final OD450 value of the test sample with that of positive control run in the same plate.

### Biochemical assays

Alanine aminotransferase activities were measured using the ALT Activity Assay Kit (Sigma-Aldrich) following the manufacturer’s instructions. Tacrolimus was measured in serum samples using the General Tacrolimus ELISA (MyBioSource, Inc., San Diego, CA) following the manufacturer’s instructions.

### HEV ORF2 immunohistochemistry

HEV ORF2 antigen was detected in FFPE liver sections by immunohistochemical staining as previously described [54].

### Terminal deoxynucleotidyl transferase dUTP nick end labeling (TUNEL) assay

Apoptotic hepatocytes were detected in FFPE liver sections using the TUNEL Assay Kit HRP-DAB (Abcam). Sections were counterstained with methyl green.

### Histopathological evaluation of liver and spleen

Liver sections were stained with Hematoxylin & Eosin (H&E) and scored blindly for inflammation by a board-certified veterinary pathologist (JMC) who enumerated foci of inflammation in ten 10X fields per section. Spleen sections were H&E stained, scanned using a Hamamatsu NanoZoomer XR digital slide scanner. The areas of lymphoid follicles in spleen tissue were measured using Hamamatsu NDP software and data exported to GraphPad Prism software for analysis.

### In situ hybridization detection of viral and cellular RNA

FFPE liver tissue sections (5μm thickness) were stained for viral and cellular RNA species by in situ hybridization (ISH) using the ViewRNA Tissue Assay (Invitrogen) according to the manufacturer’s protocol. Custom probes for detection of HEV genome RNA and gerbil CD68 mRNA were from Invitrogen. Images were acquired using an oil immersion 63X objective lens on a Zeiss CellObserver SD spinning disk confocal microscope with a Yokogawa CSU-X1 scan head.
